# A spinal manipulative therapy altered brain activity in patients with lumbar disc herniation: A resting-state functional magnetic resonance imaging study

**DOI:** 10.3389/fnins.2022.974792

**Published:** 2022-09-07

**Authors:** Ya Wen, Xiao-Min Chen, Xin Jin, Dong-Ya Ling, Shao Chen, Qin Huang, Ning Kong, Jin-Er Chai, Qing Wang, Mao-Sheng Xu, Hong-Gen Du

**Affiliations:** ^1^Department of Tuina, The First Affiliated Hospital of Zhejiang Chinese Medical University (Zhejiang Provincial Hospital of Traditional Chinese Medicine), Hangzhou, China; ^2^Department of Radiology, The First Affiliated Hospital of Zhejiang Chinese Medical University (Zhejiang Provincial Hospital of Traditional Chinese Medicine), Hangzhou, China; ^3^Department of Radiology, Changshu No. 2 People’s Hospital, The Affiliated Changshu Hospital of Xuzhou Medical University, Changshu, China

**Keywords:** lumbar disc herniation, spinal manipulative therapy, resting-state functional magnetic resonance imaging, amplitude of low-frequency fluctuations, frequency dependence

## Abstract

**Purpose:**

Lumbar disc herniation (LDH) is one of the leading causes of low-back pain and results in a series of clinical symptoms, including pain, reflex loss, and muscle weakness. Spinal manipulative therapy (SMT) can relieve pain and promote internal and external stabilization of the lumbar spine. In this study, we investigated whether the brain alterations of LDH patients with SMT were frequency-dependent based on the calculation of Amplitude of Low-Frequency Fluctuations (ALFF) and fractional ALFF (fALFF). Further, we established a cohort of LDH patients to evaluate the contribution of SMT treatments to brain functional reorganization.

**Methods:**

A total of 55 participants, including 27 LDH patients and 28 health controls (HCs), were collected. All LDH patients underwent two fMRI scans (before SMT and after the sixth SMT session). To represent LDH-related brain oscillatory activities, we calculated the ALFF and fALFF in the conventional band (0.01–0.08 Hz), the slow-4 band (0.027–0.073 Hz), and the slow-5 band (0.01–0.027 Hz). Moreover, we extracted ALFF and fALFF values in clusters with significant differences to evaluate the SMT effect.

**Results:**

Compared with HCs, the LDH patients before SMT (LDH-pre) exhibited increased fALFF in right lingual gyri in the conventional band, and showed increased fALFF in left Cerebelum_Crus1 in the slow-4 band. We further examined the abnormal brain activities changes before and after the SMT intervention. The ALFF and fALFF values of LDH-pre group were higher than those of the HCs and LDH-pos groups. After SMT, the increased ALFF and fALFF values were suppressed for patients in conventional band and slow-4 band.

**Conclusion:**

The present study characterized the altered regional patterns in spontaneous neural activity in patients with LDH. Meanwhile, SMT is an effective treatment of LDH, and we supposed that it might have been involved in modulating dysfunctional brain regions which are important for the processing of pain. The findings of the current study may provide new insights to understand pathological mechanism of LDH.

## Introduction

Lumbar disc herniation (LDH) is often associated with the protrusion or prolapse of the nucleus pulposus in the posterior spinal canal, and taking the form of a series of clinical symptoms, such as pain, reflex loss, and muscle weakness (Heliövaara, 1988). LDH is one of the most widely known causes of low-back pain (Benzakour et al., 2019). A provincial-level epidemiological investigation in China has shown that the incidence rate of LDH was 7.62% (Wang et al., 2009). Studies have reported that its prevalence is the highest between the ages of 30 and 50 years (Heliövaara, 1988; Jordan et al., 2009; Fjeld et al., 2019). Whenever feasible, most patients suffering from LDH would like to undergo conservative treatments to relieve the pain (Jung et al., 2020; Lilly et al., 2021).

Spinal manipulative therapy (SMT), which is performed by trained physicians applying a controlled force to the spine with hands or other devices, shows a higher cure and effective rate than other treatments (Li et al., 2010), and becomes the preferred complementary non-surgical conservative treatment for LDH according to the American College of Physicians (ACP) (Qaseem et al., 2017). SMT can relieve pain and promote internal and external stabilization of the lumbar spine (Field, 1998; Triano, 2001; Maigne and Vautravers, 2003; Rubinstein et al., 2019).

Resting-state functional magnetic resonance imaging (rs-fMRI) utilizes a non-destructive technique to reveal brain activities by examining the whole brain’s spontaneous fluctuations in terms of the blood oxygen level-dependent (BOLD) signal without any explicit stimulation (Biswal et al., 1995; Lee et al., 2013). Some studies have suggested that SMT might influence spontaneous brain activities in patients with low-back pain. For example, manual therapy could not only reduce clinical low-back pain but also increase functional connectivity between the salience network and the brain regions which are associated with the cognitive, affective, and sensorimotor processes of pain (Isenburg et al., 2021). Furthermore, the re-arrangement of functional connectivity could be promoted after manipulative treatments (Tramontano et al., 2020). The above studies were underpinned by functional connectivity rather than local brain alterations, it’s also important to explore whether patients have local brain dysfunctions.

Spontaneous low-frequency oscillations (LFOs) have been widely used to reveal local brain activity. Utilized in rs-fMRI analysis, the amplitude of low-frequency fluctuations (ALFF) and the fractional ALFF (fALFF) was confirmed to provide robust and reliable biomarkers for depicting regional properties of rs-fMRI data (Zang et al., 2007; Zou et al., 2008). According to Zuo et al. (2010), spontaneous LFOs can be divided into five frequency bands, including slow-6 (0–0.01 Hz), slow-5 (0.01–0.027 Hz), slow-4 (0.027–0.073 Hz), slow-3 (0.073–0.198 Hz), and slow-2 (0.198–0.25 Hz) frequency bands. And LFOs with a range of 0.01–0.073 Hz (including slow-4: 0.027–0.073 Hz and slow-5: 0.01–0.027 Hz) often embody the spontaneous activities of neurons in the gray matter (Zuo et al., 2010).

In patients with low back and leg pain, researchers have found a significant increase in ALFF in regions such as the inferior parietal lobule and medial prefrontal cortex (Zhou et al., 2018; Zhou et al., 2019). Besides, the insula, amygdala, hippocampal/parahippocampal gyrus, and thalamus have been also found sensitive to pain intensity changes (Zhang et al., 2019). Previous studies also have indicated a frequency-dependent modulation of BOLD signal oscillations in specific brain regions in patients with pain (Wang et al., 2017; Rogachov et al., 2018; Gu et al., 2019). Therefore, this research aimed to conduct the amplitude of low-frequency fluctuations (ALFF) and the fractional ALFF (fALFF) to measure local brain dysfunction in LDH patients. As far as we know, there is no previous study that has systemically assessed the frequency-specific resting-state functional changes associated with LDH patients and the effect of SMT treatments.

In this study, we utilized a cohort to evaluate the contribution of SMT treatments to LDH patients. First, we attempted to identify if there were differences in ALFF and fALFF between patients with LDH and healthy controls (HCs) in the conventional (0.01–0.08Hz), slow-4 (0.027–0.073 Hz), and slow-5 (0.01–0.027 Hz) bands. Second, according to brain regions that showed significant differences, we measured the ALFF and fALFF values of LDH patients before and after SMT, and compared them with those of HCs group. We hypothesized that altered brain regions would be frequency dependence, and dysfunctions in LDH would be improved after SMT treatments.

## Materials and methods

### Participants

We enrolled 30 LDH patients and 30 age- and sex-matched HCs from the First Clinical Medical College of Zhejiang Traditional Chinese Medical University from 24th November 2020, to 17th August, 2021. Before fMRI scanning, all patients underwent the Visual Analogue Scale (VAS) (Hawker et al., 2011), and the Chinese Short Form Oswestry Disability Index Questionnaire (C-SFODI) (Smeets et al., 2011), to evaluate their emotion status, degree of pain and daily functional activities. The inclusive criteria of LDH patients were as follows: (1) right-handed; (2) in accordance with the diagnostic criteria of LDH listed in the seventh edition of surgery; (3) aged between 20 and 60 years; (4) VAS score ≥ 30/100; (5) C-SFODI score ≥ 20%; 6) haven’t take pain therapy for at least 1 month before the enrollment. The inclusive criteria of HCs were as follows: (1) right-handed; (2) aged between 20 and 60 years; (3) no history of LDH; (4) having no pain-related treatment at least 1 month before the enrollment. The exclusive criteria for HCs were the same as LDH patients.

The exclusive criteria were as follows: (1) a history of spinal surgery or a history of severe spinal trauma; (2) bone tuberculosis, tumor, severe osteoporosis, and other orthopedics diseases; (3) combined with serious medical diseases or mental illnesses, such as those in cardiovascular and cerebrovascular, blood system and digestive system; (4) during pregnancy or breastfeeding; (5) having autoimmune diseases, allergic diseases, acute and chronic infectious diseases; (6) fMRI contraindications, such as having claustrophobia or metal implants and devices in the body; (7) fMRI examination shows free nucleus pulposus and cauda equina syndrome; (8) vision loss and vestibular dysfunction.

This study was approved by the Medical Research Ethics Committee and the Institutional Review Board of the First Clinical Medical College of the Zhejiang Traditional Chinese Medical University, and was registered in Clinical Trial Registry (No.NCT03475095). Written informed consents were obtained from all participants, and the study was conducted in accordance with the principles of the Declaration of Helsinki.

### Study design

**FIGURE 1:**
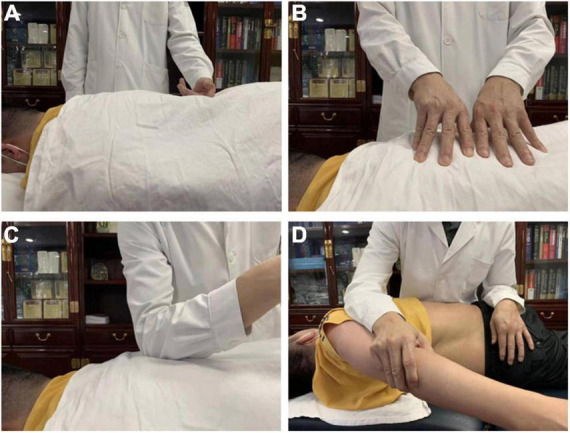
Spinal Manipulative Therapy (SMT) performed by rolling **(A)**, kneading **(B)**, plunking **(C)**, pushing **(D)** to relax the muscles in the lower back area for the purpose of alleviating pain and improving lumbar function.

All the LDH patients underwent two fMRI scans (before SMT and after the sixth SMT session), while HCs only took one scan. Each SMT session took about 25 min. All the LDH patients received SMT three times a week, and a total of six SMT sessions. There were no complications from SMT sessions in our study cohort. As shown in Figure 1, SMT includes multiple manipulations, which has been well explained in previous research (Lee et al., 2017). Specifically, we performed rolling, kneading, plunking, and pushing to relax the muscles in the lower back area, and conducted pulling-rotating maneuvers to correct disordered spine joints to alleviate pain and improve lumbar function (Figure 1).

### Quality control of spinal manipulative therapy

Requirements for physician: the SMT was operated by one physician, who had more than 5 years of massage clinical practice experience, receiving massage training which was required by the standardization of this field, and held the doctor’s qualification certification. Before conducting treatment for patients with LDH, the physician had to standardize the specific SMT operation according to the operation essentials to maintain stability in strength, shape and frequency.

Requirements for SMT environment and equipment: using a multi-functional massage bed to adjust the angle and height properly so that patients felt comfortable during the treatments. The standard treatment towel and disposable bed sheet were provided by Zhejiang hospital of traditional Chinese medicine.

### Image acquisition

All fMRI data were acquired using a 3T Siemens scanner (Verio, Siemens AG, Erlangen, Germany) with a 12-channel head coil. A sagittal T1-weighted 3D sequence with magnetization prepared rapid gradient echo (MPRAGE) was acquired for structural images. Resting-state functional images were obtained using an gradient echo-planar imaging sequence. The scanning parameters were set as follows: (1) functional MRI: 43 interleaved axial slices, matrix size = 64 × 64, field of view (FOV) = 220 mm × 220 mm, repetition time (TR) = 2,000 ms, echo time (TE) = 30 ms, flip angle = 90 degrees, slice thickness = 3.2 mm, gap = 0 (voxel size 3.4 × 3.4 × 3.2), number of volumes = 230. (2) structural MRI: Sequence = SPGR, sagittal slices, slice number = 176, matrix size = 256 × 256, FOV = 256 × 256 mm, TR/TE = 8100/3.1 ms, flip angle = 8 degrees, slice thickness = 1, gap = 0 (isotropic voxel size = 1 × 1 × 1). All participants were asked to keep their eyes closed and not to think about anything and not to fall asleep during scanning.

### Data preprocessing

The preprocessing of rs-fMRI data was conducted using RESTplus V1.25 software (Jia et al., 2019) and the steps of preprocessing include: (1) discarding the first 10 volumes to allow the signal to reach equilibrium and the participants to adapt to the scanning environment; (2) correcting for the acquisition time delayed between slices; (3) rigid-body realignment for estimation and correction of motion displacement; (4) normalizing to Montreal Neurological Institute (MNI) space by T1 new segment; (5) smoothing with a 6 mm full-width-half-maximum (FWHM) Gaussian kernel; (6) removing the linear trend; (7) regressing out Friston-24 head motion parameters (Friston et al., 1996), white matter and cerebrospinal fluid signals. After preprocessing, 3 patients and 2 HCs were excluded from the analysis because their maximum head movement exceeded 3 mm or 3 degrees.

### Amplitude of low-frequency fluctuations and the fractional amplitude of low-frequency fluctuations calculation

After data preprocessing, the time course of each voxel was transformed to the frequency using a Fast Fourier Transform (FFT) and the power spectrum was then obtained. The square root was calculated at each frequency of the power spectrum and the averaged square root obtained across a predefined frequency interval was taken as the ALFF value. fALFF is defined as the ratio of the power within a specific low-frequency range to that of the entire detectable frequency range. For standardization, the ALFF and fALFF of each voxel were divided by the global mean value of each individual for group comparison (Zang et al., 2007; Zou et al., 2008).

To determine LDH-related brain oscillatory activities, we calculated the ALFF and fALFF in the conventional band (0.01–0.08 Hz), the slow-4 band (0.027–0.073 Hz), and the slow-5 band (0.01–0.027 Hz).

### Statistical analysis

The demographics and clinical variables were analyzed using SPSS 22.0 (IBM, United States). The differences between the LDH patients and the HCs in age, years of education and clinical tests were tested with Student’s *t*-test. The sex difference was tested using the Pearson Chi-Square test.

For the ALFF and fALFF comparisons in three frequency bands, two-sample *t*-tests were performed to compare the difference between LDH patients before SMT interventions (LDH-pre) and HCs. Multiple comparison corrections were performed using Gaussian Random Field (GRF) correction with voxel-level *p* < 0.05, cluster-level *p* < 0.05. Any abnormal clusters detected by group comparisons were created as masks.

To identify the relationship between ALFF/fALFF and the SMT effect, the mean ALFF and mean fALFF values were extracted within the masks. A two-sample *t*-test was performed to compare mean ALFF and mean fALFF values between LDH-pre and HCs, LDH-pos and HCs, separately. Paired *t*-test was used to compare LDH-pos and LDH-pre. *p*-value less than 0.05 was considered significant.

### Correlation analysis

To investigate the relationship of ALFF/fALFF and VAS between before and after SMT treatments, we calculated the correlation between ALFF/fALFF changes and VAS changes. Pearson’s correlation coefficients were calculated with a significance level of *P* < 0.05.

## Results

### Demographic and clinical data

A total of 55 participants, including 27 LDH patients and 28 HCs, were selected for the present study. Table 1 demonstrated the demographic and clinical characteristics of the participants. As shown in Table 1, the age (*p* = 0.8552), years of education (*p* = 0.5691) and gender (*p* = 0.8638) were not significantly different between patients and HCs. After the sixth SMT session, VAS scores were significantly lower than VAS scores before SMT (*p* < 0.0001), and so does the C-SFODI scores (*p* < 0.0001).

**TABLE 1:** Demographic characteristics of the LDH and HC groups.

	LDH	HC	*P*-value
Participants	27	28	–
Gender (male\female)	(17\10)	(17\11)	0.8638*^a^*
Age (year)	32.2 ± 9.5	31.8 ± 8.1	0.8552*^b^*
Education (year)	16.04 ± 1.93	16.36 ± 2.20	0.5691*^b^*
VAS scores (pre\pos)	(5.6 ± 2.1\1.7 ± 1.1)	–	<0.0001*^c^*
C-SFODI scores (pre\pos)	(26.9 ± 8.1\18.7 ± 5.4)	–	<0.0001*^c^*

*^a^*χ^2^-test;

*^b^*Two sample t-test;

*^c^*Paired t-test between LDH patients before (LDH-pre) and after SMT treatment (LDH-pos).

LDH, Lumbar disc herniation; HC, health control; VAS, Visual Analogue Scale; C-SFODI, Chinese Short Form Oswestry Disability Index Questionnaire.

### Amplitude of low-frequency fluctuations and the fractional amplitude of low-frequency fluctuations differences between lumbar disc herniation-pre patients and health controls

**FIGURE 2:**
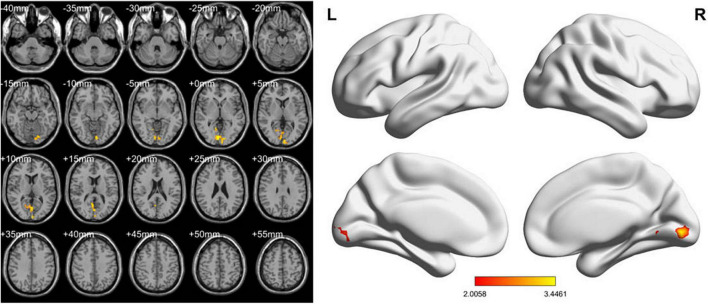
ALFF values using two-sample *t*-tests in the conventional band between LDH-pre patients and HCs. Increased ALFF values have been shown in the right lingual gyri in LDH-pre group compared with HCs. (GRF correction, voxel *p* < 0.05 and cluster *p* < 0.05). Color bar indicates the *t* score (L, left; R, right).

**FIGURE 3:**
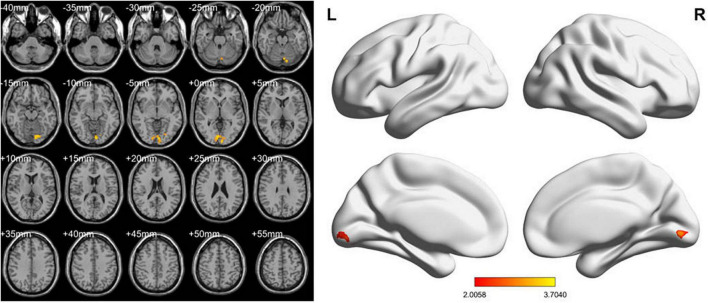
fALFF values using two-sample *t*-tests in slow-4 band between LDH-pre patients and HCs. Increased fALFF values have been shown in the left Cerebelum_Crus1 in LDH-pre group compared with HCs (GRF correction, voxel *p* < 0.05 and cluster *p* < 0.05). Color bar indicates the *t* score (L, left; R, right).

In the conventional band, compared with the HCs, the LDH-pre patients exhibited increased fALFF in the right lingual gyri (Table 2 and Figure 2). In the slow-4 band, the LDH-pre patients showed increased fALFF in left Cerebelum_Crus1 (Table 2 and Figure 3). In the slow-5 band, there were no brain regions with significant differences between the two groups.

**TABLE 2:** Regions showing abnormal ALFF and fALFF in LDH-pre compared with HCs.

	Brain area	Voxel size	MNI coordinates	Peak *t*-value
Conventional band				
mALFF	–	–	–	–
mfALFF	Lingual_R	245	9, –84, –3	3.4461
Slow-4 band				
mALFF	–	–	–	–
mfALFF	Cerebelum_Crus1_L	166	–12, –81, –18	3.704
Slow-5 band				
mALFF	–	–	–	–
mfALFF	–	–	–	–

MNI, Montreal Neurological Institute; Lingual_R, right lingual gyri; Cerebelum_Crus1_L, left Cerebelum_Crus1; LDH-pre, Lumbar disc herniation patients before SMT treatment.

No regions showed significant differences in ALFF between LDH-pre patients and HCs in every band.

### Evaluation of spinal manipulative therapy effect in right lingual gyri

**FIGURE 4:**
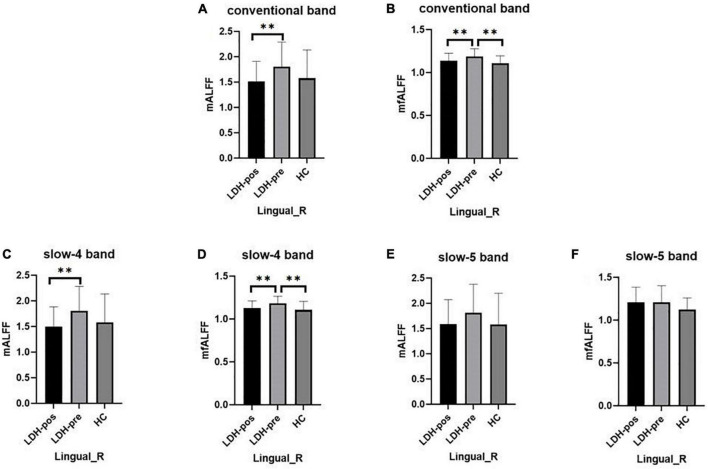
The mean ALFF and fALFF values of signals extracted from right lingual gyri of each participant. The LDH patients and HCs were compared using two-sample *t*-tests, and LDH patients before and after SMT were compared by paired *t*-tests. ^**^ Indicates significance level at *p* < 0.01 (two -tailed). **(A,B)** The mALFF value **(A)** and mfALFF value **(B)** in the conventional band from the right lingual gyri of the HC, the patients with LDH before (LDH-pre) and after the SMT treatments (LDH-pos). **(C,D)** The mALFF value **(C)** and mfALFF value **(D)** in the slow-4 band from the from the same brain region of the three group. **(E,F)** The mALFF value **(E)** and mfALFF value **(F)** in the slow-5 band.

We extracted the mean ALFF and fALFF values within the right lingual gyri which showed significant abnormal alterations between LDH-pre and HCs groups. In the conventional band, the results revealed the decreased ALFF in LDH-pos patients compared with LDH-pre patients (*p* = 0.0029) and closed to HCs (*p* = 0.6025) (Table 3 and Figure 4A). A significant difference of fALFF was also observed between LDH-pos and LDH-pre (*p* = 0.0035) (Figure 4B). After the SMT treatments, no significant difference was found in fALFF between LDH-pos and HCs (*p* = 0.1896) (Table 3 and Figure 4B). Furthermore, we also found the LDH-pre exhibited significantly higher fALFF than HCs (*p* = 0.0011) (Table 3 and Figure 4B). In the slow-4 band, the LDH-pos showed decreased ALFF compared with LDH-pre (*p* = 0.0018), and no significant difference was found in ALFF between LDH-pos and HCs (*p* = 0.5066) (Table 4 and Figure 4C). Similar tendencies were also found in fALFF. LDH-pre exhibited significantly higher fALFF than HCs (*p* = 0.0025) (Table 4 and Figure 4D). A significant difference was also observed between LDH-pos and LDH-pre (*p* = 0.0032) groups (Table 4 and Figure 4D). After the SMT treatments, we did not observe any significant fALFF difference between LDH-pos and HCs (*p* = 0.4277) (Table 4 and Figure 4D). In the slow-5 band, no significant difference has been found in the ALFF and fALFF in every group comparison (Table 5 and Figures 4E,F).

**TABLE 3:** Group differences in ALFF and fALFF in conventional band.

Coordinate	Brain region	Metrics	Contrast	*t*-value	*P*-value
9, –84, –3	Lingual_R	ALFF	LDH-pre—HC	1.628	0.1094
			LDH-pos—LDH-pre	3.291	0.0029
			LDH-pos—HC	0.524	0.6025
		fALFF	LDH-pre—HC	3.461	0.0011
			LDH-pos–LDH-pre	3.209	0.0035
			LDH-pos—HC	1.329	0.1896
–12, –81, –18	Cerebelum_Crus1_L	ALFF	LDH-pre—HC	1.556	0.1256
			LDH-pos—LDH-pre	3.476	0.0018
			LDH-pos—HC	0.508	0.6136
		fALFF	LDH-pre—HC	3.295	0.0018
			LDH-pos—LDH-pre	2.810	0.0093
			LDH-pos—HC	1.233	0.2231

Lingual_R, right lingual gyri; Cerebelum_Crus1_L, left Cerebelum_Crus1; LDH-pre, Lumbar disc herniation patients before SMT treatment; HC, healthy control; LDH-pos, Lumbar disc herniation patients after SMT treatment.

**TABLE 4:** Group differences in ALFF and fALFF in slow-4 band.

Coordinate	Brain region	Metrics	Contrast	*t*-value	*P*-value
9, –84, –3	Lingual_R	ALFF	LDH-pre—HC	1.600	0.1155
			LDH-pos—LDH-pre	3.479	0.0018
			LDH-pos—HC	0.669	0.5066
		fALFF	LDH-pre—HC	3.171	0.0025
			LDH-pos—LDH-pre	3.253	0.0032
			LDH-pos—HC	0.7992	0.4277
–12, –81, –18	Cerebelum_Crus1_L	ALFF	LDH-pre—HC	1.743	0.0871
			LDH-pos—LDH-pre	3.904	0.0006
			LDH-pos—HC	0.5701	0.5710
		fALFF	LDH-pre—HC	3.881	0.0003
			LDH-pos—LDH-pre	3.436	0.0020
			LDH-pos—HC	1.070	0.2895

Lingual_R, right lingual gyri; Cerebelum_Crus1_L, left Cerebelum_Crus1; LDH-pre, Lumbar disc herniation patients before SMT treatment; HC, healthy control; LDH-pos, Lumbar disc herniation patients after SMT treatment.

**TABLE 5:** Group differences in ALFF and fALFF in slow-5 band.

Coordinate	Brain region	Metrics	Contrast	*t*-value	*P*-value
9, –84, –3	Lingual_R	ALFF	LDH-pre—HC	1.462	0.1495
			LDH-pos—LDH-pre	1.933	0.0642
			LDH-pos—HC	0.040	0.9684
		fALFF	LDH-pre—HC	1.895	0.0635
			LDH-pos—LDH-pre	0.053	0.9582
			LDH-pos—HC	1.927	0.0593
–12, –81, –18	Cerebelum_Crus1_L	ALFF	LDH-pre—HC	1.011	0.3166
			LDH-pos—LDH-pre	1.641	0.1127
			LDH-pos—HC	0.099	0.9217
		fALFF	LDH-pre—HC	0.7179	0.4760
			LDH-pos—LDH-pre	0.6177	0.5421
			LDH-pos—HC	1.305	0.1974

Lingual_R, right lingual gyri; Cerebelum_Crus1_L, left Cerebelum_Crus1; LDH-pre, Lumbar disc herniation patients before SMT treatment; HC, healthy control; LDH-pos, Lumbar disc herniation patients after SMT treatment.

### Evaluation of spinal manipulative therapy effect in left Cerebelum_Crus1

**FIGURE 5:**
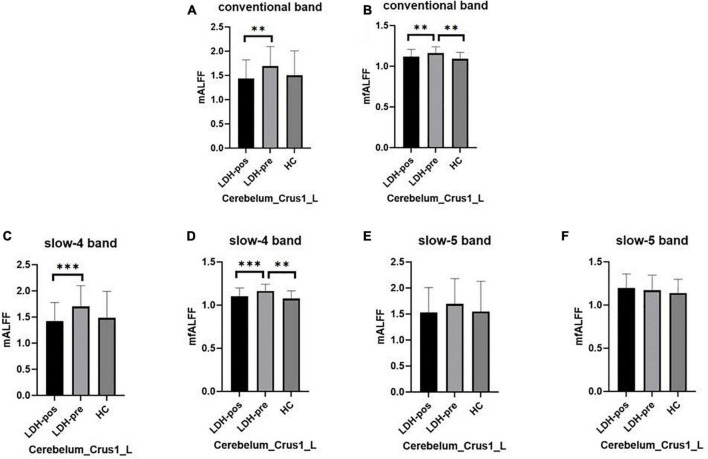
The mean ALFF and fALFF values of signals extracted from left Cerebelum_Crus1 of each participant. The LDH patients and HCs were compared using two-sample *t*-tests, and LDH patients before and after SMT were compared by paired *t*-tests. ^**^ Indicates significance level at *p* < 0.01 (two -tailed), ^***^ Indicates significance level at *p* < 0.001 (two -tailed). **(A,B)** The mALFF value **(A)** and mfALFF value **(B)** in the conventional band from left Cerebelum_Crus1 of the HC, the patients with LDH before (LDH-pre) and after the SMT treatments (LDH-pos). **(C,D)** The mALFF value **(C)** and mfALFF value **(D)** in the slow-4 band from the from the same brain region of the three group. **(E,F)** The mALFF value **(E)** and mfALFF value **(F)** in the slow-5 band.

We extracted the mean ALFF and fALFF values within left Cerebelum_Crus1 which showed significant abnormal changes between LDH-pre patients and HCs. In the conventional band, the LDH patients showed decreased ALFF after SMT treatments (*p* = 0.0018), and the ALFF values were not significant compared with the HCs group (*p* = 0.6136) (Table 3 and Figure 5A). Compared with the HCs group, the LDH-pre group showed increased fALFF in the left Cerebelum_Crus1 (*p* = 0.0018). After SMT, the LDH-pos showed decreased fALFF compared with LDH-pre (*p* = 0.0093) (Table 3 and Figure 5B). In the slow-4 band, the LDH patients showed decreased ALFF and fALFF after SMT treatments (*p* = 0.0006 and *p* = 0.0020) (Table 4 and Figures 5C,D) compared with the LDH-pre group. Meanwhile, a significant fALFF difference was also observed between LDH-pre and HCs (*p* = 0.0003). In the slow-5 band, no significant difference was observed in ALFF and fALFF (Table 5 and Figures 5E,F).

### Correlations between Amplitude of low-frequency fluctuations/fractional amplitude of low-frequency fluctuations and visual analogue scale in lumbar disc herniation

No significant correlation was found between the ALFF/fALFF changes and VAS changes.

## Discussion

In this study, we compared the ALFF and fALFF in three bands between LDH patients and HCs, and the regions that showed functional alternations were mainly found in right lingual gyri the and left Cerebelum_Crus1. Subsequently, we extracted the ALFF and fALFF values of LDH-pre patients, LDH-pos patients, and HCs over the right lingual and left Cerebelum_Crus1. After SMT, the ALFF and fALFF of LDH patients was decreased compared with that of the LDH-pre group. Our study controlled some potential confounding factors, such as age, education level, and excluded participants with a confirmed psychiatric diagnosis. The finding of this study are still limited to elucidate the neurobiological basis of LDH associated with alterations in spontaneous activity, and there is still a need for more evidence that could present the relationship between altered brain functions resulted from pain and the modulation of SMT in LDH patients.

Compared with the HCs group, we found that the LDH patients showed increased fALFF in the right lingual gyri extending to calcarine in the conventional band. The lingual gyri is mainly located in the visual cortex and sends sensory information through sensory afferents to the thalamus, amygdala, and hippocampus (Gorman et al., 2000). One study have found that visceral pain was associated with the hippocampus, fusiform gyrus, striatum, occipital cortex, insula, and amygdala (Schmidt et al., 2022). Furthermore, there is growing evidence that the visual cortex has also been involved in the processing of pain signals (Liu et al., 2019; Wei et al., 2019; Bush et al., 2021). We compared the alterations of lingual gyri in LDH-pre and HCs, LDH-pre and LDH-pos respectively, and the results presented that both ALFF and fALFF in right lingual gyri were sensitive to SMT treatments in conventional and slow-4 bands.

The cerebellum may play an essential role in integrating functions, including memory, associative learning, and motor control, and it participates actively in sensory processing, such as nociception (Schmahmann, 1991; Schmahmann, 1997; Ito, 2006). Thus, the cerebellum has appeared to be involved in the process of integrating motor, sensory, autonomic, and cognitive responses to environmental stimuli, including acute and chronic pain (Borsook et al., 2008). We found dysfunction of Cerebelum_Crus1 in the slow-4 band. Meanwhile, the ALFF and fALFF values in Cerebelum_Crus1 had a significant difference between LDH-pre and LDH-pos group both in the conventional and slow-4 band, suggesting that Cerebelum_Crus1 might be sensitive to changes in pain intensity. A previous study has proposed a biopsychosocial mechanism of manipulative therapy treatment which referred to a post-treatment reduction in both clinical pain and fear of “back-straining” exercises, relating decreased BOLD response to various brain regions that are associated with emotion, cognition, and pain perception (Ellingsen et al., 2018).

Until now, SMT has been utilized to restore the structure of disordered spines and unstable spinal balance, improve the physiological properties of muscles, moderate the internal stress of intervertebral discs, and alleviate intervertebral disc compression of the nerve root, therefore, it could reach the goal of stopping pain and restoring the function of the local spinal column (Maigne and Vautravers, 2003; Bredow et al., 2016). A relevant systemic review has shown that SMT had a favorable effect on alleviating pain and speeding up the recovery, compared with other interventions for LDH patients (Maxwell et al., 2020). A prospective observational cohort study with a 1-year follow-up also has indicated that a large percentage of acute and chronic LDH patients treated with SMT have been reported clinically relevant improvement (Leemann et al., 2014). Therefore, SMT provides an effective conservative treatment for patients with LDH and avoids the risk of surgery to a great extent.

However, the effectiveness of SMT might be affected by other factors. By comparing standard care plus SMT and standard medical care which included drug treatment, and maintaining normal daily activity levels. One study has shown no significant differences between groups after 2 weeks and 6 months (Juni et al., 2009). While Goertz et al. (2013) have reported that adding SMT can improve significantly pain and disability in 2–4 weeks. These inconsistent results are probably explained by differences in the study designs. Furthermore, the number of SMT sessions has been not yet standardized among different studies. A higher frequency of SMT sessions seems to observe a significant effect of SMT (Goertz et al., 2013; Schneider et al., 2015), compared to the lower frequency of SMT intervention (Juni et al., 2009). In addition, some potential effect could not be ruled out, such as the patient expectations regarding the treatment (Rubinstein et al., 2019) and the severity level of the patients (Paige et al., 2017).

The combination of SMT and fMRI provides a new perspective for us to study the pathological mechanism of SMT. However, some limitations need to be considered in our study. Firstly, due to the relative strict exclusion criteria, i.e., only patients who were in accordance with the diagnostic criteria of LDH listed in the seventh edition of surgery, and didn’t accept pain therapy for at least 1 month before the enrollment could be included in the current research, the sample size was relatively small, which lead to a small effect size. To facilitate the later studies, we shared the original ALFF and fALFF data and uncorrected *t* maps. Second, considering the potential risk of comorbidity, the associations between altered spontaneous brain activity and the effect of comorbidity are unclear, and the underlying mechanisms of disrupted regional activities in LDH need to be further studied. Our analysis could be seen as an exploratory study, which provides the primary evidence for brain alterations of LDH patients during resting-state. Further, multimodal imaging approaches should be applied to elucidate the relationship between ALFF or fALFF and the neural mechanism of LDH.

## Conclusion

The ALFF and fALFF analysis deployed in this study revealed the altered regional activities in multiple frequency bands in LDH patients. We found that the abnormalities of LFO amplitude in LDH existed frequency dependent. Additionally, we assessed the effect of SMT treatments. Our findings may provide a practical rationale and mechanism for studying the frequency-dependence of BOLD oscillations from the perspective of SMT.

## Data availability statement

The raw data supporting the conclusions of this article will be made available by the authors, without undue reservation.

## Ethics statement

The studies involving human participants were reviewed and approved by the Medical Research Ethics Committee and the Institutional Review Board of the First Clinical Medical College of the Zhejiang Traditional Chinese Medical University. The patients/participants provided their written informed consent to participate in this study.

## Author contributions

H-GD and M-SX designed the study. YW collected the data. X-MC analyzed the data. YW wrote the first version. XJ, D-YL, SC, and QH revised the manuscript and all other authors added their comments. All authors approved the manuscript.
